# circPOLA2 promotes proliferation, invasion, migration, and epithelial-mesenchymal transition in breast cancer via the miR-1224–5p/HMGA2 axis

**DOI:** 10.1016/j.clinsp.2025.100653

**Published:** 2025-04-23

**Authors:** XinYan Xu, Jie Li, RuiJuan Li, YanFang Tan, ZhiBing Lu

**Affiliations:** a Department of Oncology, Pingxiang People's Hospital, Pingxiang City, Jiangxi Province, PR China; b Department of Breast, Pingxiang People's Hospital, Pingxiang City, Jiangxi Province, PR China

**Keywords:** circPOLA2 miR-1224–5p HMGA2 Breast Cancer Epithelial-Mesenchymal Transition Metastasis

## Abstract

**Objective:**

This study aimed to investigate the carcinogenic role of circPOLA2 in Breast Cancer (BC) and reveal its potential mechanism as a competitive endogenous RNA.

**Methods:**

Differentially expressed circRNAs, miRNAs, and mRNAs in BC tissues and cell lines were screened and analyzed by RT-qPCR. The interaction among circPOLA2, miR-1224–5p, and HMGA2 was tested using dual luciferase reporter assay and RNA pull-down assay. Cell proliferation was detected by MTT and colony formation assay, apoptosis was detected by flow cytometry, migration, and invasion was detected by Transwell assay, and EMT-related proteins were detected by Western blot.

**Results:**

circPOLA2 and HMGA2 levels were elevated in BC, while miR-1224–5p level was reduced. Knocking down circPOLA2 decreased the expression of HMGA2 by elevating miR-1224–5p expression. Knocking down circPOLA2 or HMGA2 or elevating miR-1224–5p reduced the proliferative, migratory, invasive, and anti-apoptotic capacities of BC cells.

**Conclusion:**

Knockdown of circPOLA2 inhibits BC cell proliferation, migration, and invasion and delays BC tumor progression by regulating the miR-1224–5p/HMGA2 axis, providing a new strategy and target for therapeutic intervention in BC.

## Highlights

• Upregulated circPOLA2 in BC is associated with poor prognosis.
• circPOLA2 triggers BC cell malignant phenotype.
• circPOLA2 directly interacts with miR-1224–5p in cells.
• Depleting miR-1224–5p reverses si-circPOLA2-mediated inhibition of malignant behavior in BC cells.

## Introduction

In female malignant tumor patients, Breast Cancer (BC) is the most prevalent, with high mortality rates^1^^,^^2^ Annually, over 1.3 million new cases of BC are diagnosed globally, with more than 500,000 deaths^3^^,^^4^ Current treatments for BC include radiotherapy and chemotherapy, but a lack of targeted therapy adversely affects recurrence and survival rates. Therefore, understanding the pathogenesis of BC and exploring new treatment strategies are crucial for improving BC prognoses.

CircRNAs are non-coding RNAs that are single-stranded and formed through back-splicing, known for their high stability and specificity^5^ Due to the absence of free 5′ or 3′ ends, circRNAs exhibit greater resistance to RNAse R, resulting in increased stability and making them more challenging to degrade than linear RNA^6^ circRNAs play roles in the biological processes of human cancers, including BC^7^ Meanwhile, circRNAs can exert their biological activities as competing endogenous RNAs (ceRNAs) for microRNAs (miRNAs), regulating tumor development by mediating drug resistance^8^ For example, hsa_circ_0061825 promotes BC cell proliferation, invasion, and Epithelial-Mesenchymal Transition (EMT) by targeting miR-326/TFF1 signaling^9^ hsa_circRNA_0006528 functions as a ceRNA to promote BC progression through the miR-7–5p/MAPK/ERK pathway^10^ Studies have identified circ_POLA2 as an oncogenic circRNA in lung and cervical cancers. circPOLA2 may interact with the miR-326/GNB1 axis to increase lung cancer cell stemness^11^ circPOLA2 is up-regulated in cervical cancer and promotes cancer progression through competitively binding to miR-326^12^ Moreover, circPOLA2 is identified as an oncogene in colon cancer^13^ endometrial cancer^14^ and glioblastoma^15^ This suggests, in part, that, along with other circRNAs, circPOLA2 may be a specific target for tumor development and therapy, and moreover gives rise to an in-depth exploration of the role of circPOLA2 in BC. Although studies on circPOLA2′s expression and mechanism in breast cancer are absent, it is possible that circPOLA2 could be crucial in breast cancer by influencing miRNAs, based on the general roles and mechanisms of circRNAs. The unexplored and unique attributes of circPOLA2 may present it as a promising new target. miRNAs are small non-coding RNAs that bind to complementary sequences in the 3′-UTR of target genes. Abnormal expression of miRNAs is closely related to tumor development and can affect tumor cell biological behaviors, such as proliferation, apoptosis, and invasion^16^, ^17^, ^18^, ^19^ MiR-1224–5p is involved in EMT in osteosarcoma^20^ Regulating miR-1224–5p provides a promising target for polycystic ovary syndrome^21^ Additionally, miR-1224–5p suppresses colorectal tumor proliferation, metastasis, and glycolysis^22^ Although miR-1224–5p is closely associated with the development of most cancers, it is still poorly explored in BC. Wang et al. showed that miR-1224–5p is a down-regulated miRNA in ovarian cancer, which is involved in epithelial-mesenchymal transition in BC cells[23] and thus regulates cancer progression. High Mobility Group A2 (HMGA2) is a predicted target protein of miR-1224–5p As a transcriptional regulator, HMGA2 contains structural DNA-binding domains. Abnormal expression of HMGA2 is detected in cancer development, and this protein is an upstream mediator of cancer apoptosis, proliferation, invasion, metastasis, and treatment resistance^24^^,^^25^ HMGA2 also controls gene networks involved in the key processes of BC^26^ HMGA2 mRNA levels in tumors are significantly higher than those in paracancerous tissues of BC patients^27^ and play a role in tumor progression and drug resistance by affecting processes including tumor growth, infiltration, metastasis, and apoptosis. However, whether HMGA2 can be directly targeted by miR-1224–5p and its role in BC therapy have not been investigated.

The aim of this study was to investigate whether circPOLA2 affects BC cell biological function by influencing gene expression. Here, with the help of multiple experimental methods and bioinformatics software, the authors explored the interactions between circPOLA2, miR-1224–5p, and HMGA2, as well as the mechanism of action by which circPOLA2 regulates the miR-1224–5p/HMGA2 axis to promote the development of BC cells.

## Materials and methods

### Clinical samples

Sixty BC patients in Pingxiang People's Hospital were included, from whom BC tumor tissues were obtained, as well as adjacent non-tumor tissues (3 cm from tumor margin). Neither radiotherapy nor chemotherapy was administered to the patients before surgery. Specimens were immediately frozen in liquid nitrogen and stored at −80 °C. Pingxiang People's Hospital Ethics Committee granted approval for this study (n° 202007JS6). Informed consent was given to all patients.

### Cell culture

BC cells (MCF-7, MDA-MB-231, and MDA-MB-468) and normal breast epithelial cells MCF-10A were obtained from the National Collection of Authenticated Cell Cultures (Shanghai, China). BC cells were put in DMEM (Gibco, USA) composed of 10 % FBS (Gibco) and 1 % penicillin/streptomycin (Beyotime, Shanghai, China) at 37 °C and 5 % CO_2_. The culture medium for MCF-10A cells was DMEM/F12 (Gibco) composed of 5 % horse serum medium (Gibco), 10 μg/mL insulin (Sigema, Germany), 0.5 μg/mL hydrocortisone (Sigema), 100 ng/mL cholera toxin (Sigema), and 20 ng/mL EGF (Sigema).

### Proliferation assay

MCF-7 cells were transfected for 24 h, 48 h, and 72 h and added with 10 μL MTT solution (Vazyme, Nanjing, China) at 37 °C for 4 h. After removing the supernatant, the crystals were dissolved by adding 100 μL DMSO (Sigma-Aldrich, USA). On a microplate reader, measurements of OD values at 490 nm were conducted. Cell viability = (experimental group OD - blank group OD)/(control group OD - blank group OD) × 100 %.

### Actinomycin D (Act D) assay

MCF-7 cell culture medium was added with 5 µg/mL Act D (Sigma) and collected at the specified time point. Total RNA was extracted to study circPOLA2 expression by RT-qPCR.

### RNAse R assay

MCF-7 cells were digested with 3 U/μg RNAse R reagent (Epicentre, USA) at 37 °C for 15 min, followed by RNA purification using the RNeasy MinElute Cleanup kit (Qiagen, Germany). circPOLA2 and linear POLA2 were then studied using RT-qPCR.

### Cell transfection

circPOLA2 small interfering RNA (si-circPOLA2), HMGA2-overexpressed plasmid (pcDNA3.1-HMGA2), miR-1224–5p-mimic/inhibitor, and negative controls (si-NC, pcDNA3.1-NC, mimic-NC, and inhibitor-NC) were produced by GenePharma (Shanghai, China). When MCF-7 cells reached 60 %‒70 % confluence, the above oligonucleotides and plasmids were transfected instantaneously using Lipofectamine 3000 (Invitrogen, USA). Cells were collected 24 h later, and the transfection efficiency was assessed using RT-qPCR or Western blot.

### RT-qPCR

RNA was extracted from tissues and cells using TRIzol® reagents (Invitrogen). The RNA quality was then measured by Nanodrop 2000. miRNA cDNA was produced using miRNA reverse transcription kit (TaKaRa), and mRNA/circRNA cDNAs were synthesized using PrimeScript™RT Reagent kit (TaKaRa). Three replicates of RT-qPCR were conducted using TB Green® Fast qPCR Mix (TaKaRa), with U6 and GAPDH as endogenous reference genes. Gene expression was calculated by the 2^-ΔΔCt^ method. Primers are found in Table 1.

**Table 1:** Primers.

Name	Primer sequences (5′‒3′)
circPOLA2	Forward: TTTCAAGCAGTGTCTACGAACA
	Reverse: AGAACGTCCTGCTTCCCAAA
POLA2	Forward: ACCCAGAGGAGCTACTACCC
	Reverse: TCCCACCTGCCCTTTGGTA
miR-1224–5p	Forward: GCGGCGGGTGAGGACTCGGGAG
	Reverse: ATCCAGTGCAGGGTCCGAGG
HMGA2	Forward: AGCCCTCTCCTAAGAGACCC
	Reverse: GCAAGGCAACATTGACCTGAG
GAPDH	Forward: CAAATTCCATGGCACCGTCA
	Reverse: GATGGCATGGACTGTGGTCA
U6	Forward: CTCGCTTCGGCAGCACA
	Reverse: AACGCTTCACGAATTTGCGT

### Apoptosis assay

The apoptosis rate was assessed by the FITC Annexin V Apoptosis Detection Kit (BD Biosciences, USA). MCF-7 cells were suspended and rinsed twice with pre-cooled PBS. Cells were re-suspended in 500 μL 1 × binding buffer and mixed with 5 μL Annexin V-FITC and 5 μL PI solutions, respectively, for 15 min. The proportion of apoptotic cells was measured on the FACScan flow cytometer (BD Biosciences).

### Luciferase activity analysis

StarBase 3.0 (http://starbase.sysu.edu.cn/) predicted binding sites between miR-1224–5p and HMGA2/circPOLA2. miRBase (https://www.mirbase.org/) and DIANA-miRPath (https://dianalab.e-ce.uth.gr/) predicted the targeting relationship between miR-1224–5p and HMGA2^28^ GenePharma synthesized wild-type and mutant-circPOLA2 fragments containing miR-1224–5p binding sites, as well as wild-type and mutant-HMGA2 fragments. pmirGLO vector (Promega, USA) was cloned with the fragments to obtain WT-circPOLA2 and MUT-circPOLA2, as well as WT-HMGA2 and MUT-HMGA2. Using Lipofectamine® 3000 (Invitrogen), the reporter was co-transfected into MCF-7 cells containing miR-1224–5p mimic or mimic-NC. Cells were incubated at 37° and 5 % CO_2_ for 48 h and checked for luciferase activity using a dual luciferase reporter gene assay system (Promega) in combination with Synergy 2 Multidetector Microplate Reader (BioTek Instruments. USA).

### Colony-forming ability assay

MCF-7 cells (1 × 10^3^ cells/well) were put into a 6-well plate and cultured in DMEM containing 10 % FBS in a 37° incubator. Two weeks later, the colonies were rinsed with PBS (twice) and fixed in 4 % paraformaldehyde for 20 min before 0.1 % crystal violet staining for 30 min. The number of colonies was calculated.

### Western blot

MCF-7 cells were rinsed with pre-cooled PBS twice, and the lysis buffer (Beyotime) was added for 20 min. Following protein concentration detection using Bradford assay (Bio-Rad, USA), the proteins were then separated by 15 % SDS-PAGE and transferred to PVDF (Beyotime) membranes. Blocked at room temperature with 5 % skim milk powder for 1 h, the membranes were then rinsed 3 times with TBST (Vazyme). The primary antibodies including E-cadherin (#3195, CST, USA), N-cadherin (#13,116, CST), Vimentin (#5741, CST), HMAG2 (#ab246513, Abcam, USA), GAPDH (#ab246513, Abcam) were incubated overnight at 4 °C. After three rounds of TBST washing, the secondary antibody (CST) bound to horseradish peroxidase was incubated at 37 °C for 1 h, and then bands were developed using an ECL kit (Gltrassignal, China).

### Migration and invasion tests

Cell migration and invasion experiments were conducted with 8 μm pore size (Corning, USA), and Transwell chambers (BD Biosciences, USA) were coated with matrigel (BD Biosciences). Matrigel was not needed in the migration test. MCF-7 cells (4 × 10^4^ migration/8 × 10^4^ invasion) were re-suspended in the upper cavity containing 200 μL serum-free medium, and 600 μL medium containing 20 % FBS was added in the lower cavity. After 24 h of culture, cells fixed with 4 % paraformaldehyde in the lower chamber were imaged with a microscope (Olympus, Tokyo, Japan), followed by analysis with ImageJ software.

### RNA pull-down assay

Biotin-labeled wild-type miR-1224–5p (Bio-miR-1224–5p-WT) and mutated miR-1224–5p (Bio-miR-1224–5p-MUT) probes were transfected into MCF-10A cells. After lysis, the products were incubated with streptavidin magnetic beads (Invitrogen) at 4 °C for 4 h and washed 5 times. RNA complexes were extracted and purified with TRIzol reagent (Sigma-Aldrich) to measure target gene expression by RT-qPCR.

### Xenograft experiment

All animals were programmed with the provisions of the Animal Committee of Pingxiang People's Hospital (n° 2020JS147). MCF-7 cells (5 × 10^6^) were transfected with si-circPOLA2 or si-NC for 24 h, re-suspended in 100 μL PBS, and then injected into the mammary fat pad of 4-week-old female BALB/c nude mice (Shanghai Lab. Animal Research Center), with 5 mice per group. Tumor diameter was measured with a caliper every 4 days. All mice were euthanized at week 4 after the final measurement. The tumors were weighed and photographed. Tumor volume = 1/2 × length × width^2^. Animal studies were performed in compliance with the ARRIVE guidelines.

### Immunohistochemical staining

Mouse tumors were fixed with 10 % neutral formaldehyde and then continuously sliced at 2 μm using a microtome (RM2016, leicaSOLMS, Germany). Slices were dewaxed with xylene and graded with ethanol, followed by incubation in PBS solution containing 5 % FBS and 0.3 % Triton X-100 (Beyotime) at room temperature for 1 h. Next, HMAG2 (#ab246513, Abcam) was added at 4 °C overnight, and the secondary antibody IgG (#ab124055, Abcam) was reacted at room temperature for 1 h before adding DAB substrate (Vector Labs, Burlingame, CA, USA). Slices were re-stained with hematoxylin for 2 min and observed under a microscope.

### Statistical analysis

Statistical analysis was done using GraphPad Prism 8 (GraphPad Software, USA) and SPSS Statistics 20 (IBM, USA). The data were expressed as mean ± Standard Deviation (SD), and each experiment was biologically replicated at least three times. Student's *t*-test was applied to compare two-group differences, while one-way ANOVA was utilized to compare multiple-group differences. The correlation between circPOLA2 expression and clinicopathological information of patients was evaluated by χ^2^ test. Kaplan-Meier and Log-rank test evaluated the overall survival of patients; * *p* < 0.05 was considered statistically significant.

## Results

### Upregulated circPOLA2 in BC is associated with poor prognosis

**Fig. 1:**
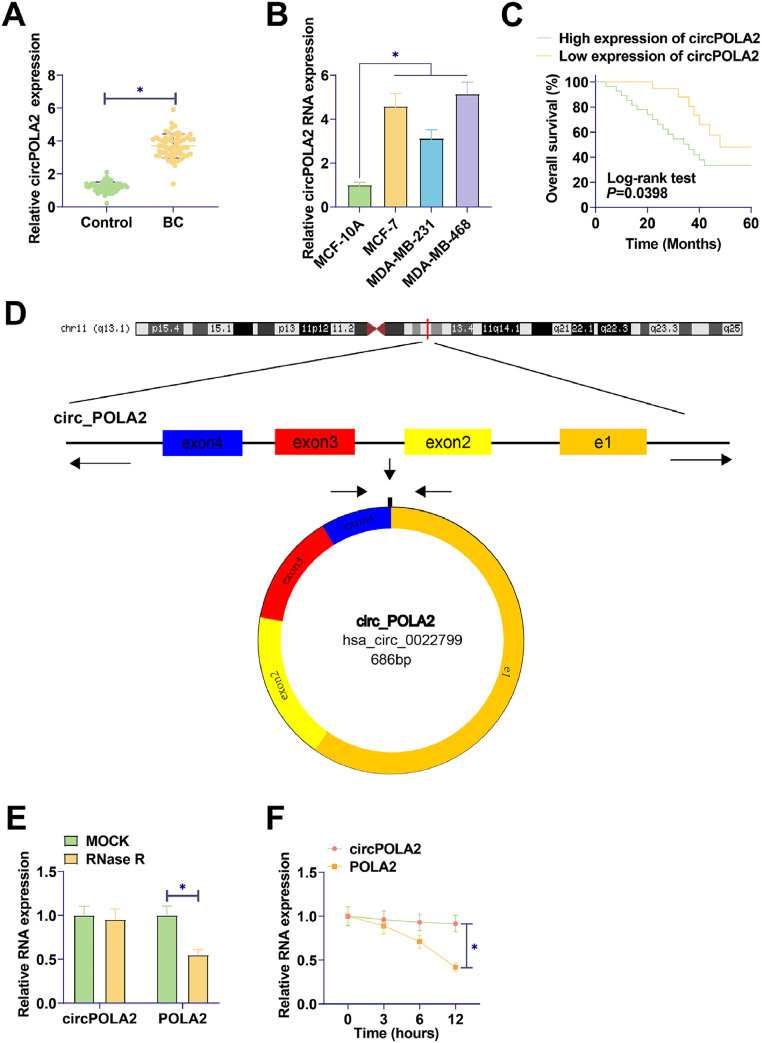
circPOLA2 is upregulated in BC and is associated with malignant progression and poor prognosis. (A) RT-qPCR measured circPOLA2 levels in tissues; (B) RT-qPCR measured circPOLA2 in MCF-7, MDA-MB-231, MDA-MB-468, and MCF-10A; (C) Kaplan-Meier analyzed the correlation between circPOLA2 expression and overall survival; (D) circPOLA2 structure; (E) RNAse R treatment verified the stability of circPOLA2; (F) Act D treatment verified the stability of circPOLA2. Data are expressed as mean ± SD (*n* = 3), * *p* < 0.05.

RT-qPCR was performed on 60 pairs of BC tissues and non-tumor tissues. circPOLA2 in tumor tissues of BC patients was higher than that in non-tumor tissues (Fig. 1A). circPOLA2 was upregulated in BC cell lines (MCF-7, MDA-MB-231, and MDA-MB-468) compared to MCF-10A cells (Fig. 1B). An investigation of the relationship between circPOLA2 expression and clinical characteristics among BC patients was conducted. With reference to circPOLA2 median expression, BC patients were categorized into the high-expression group and low-expression group. The expression of circPOLA2 at high levels was significantly related to TNM stage, tumor size, and distant metastasis, but not to age or lymph node metastasis (Table 2). Kaplan-Meier analysis showed that BC patients with high circPOLA2 expression had shorter overall survival (Fig. 1C). This suggests that circPOLA2 may be a poor prognostic factor. To characterize the circRNA transcript, a bioinformatic locus (http://www.circbase.org/) and Primer were utilized to determine the stability of circPOLA2 (Fig. 1D). circPOLA2 was resistant to RNAse R, while POLA2 was rapidly digested by RNAse R (Fig. 1E). Act D results showed that circPOLA2 was more stable than POLA2 (Fig. 1F).

**Table 2:** Correlation between circPOLA2 expression and clinical variables in BC patients.

Characteristics	N	circPOLA2 low expression (*n* = 30)	circPOLA2 high expression (*n* = 30)	p-value
Age				
< 50	33	16	17	0.7952
≥ 50	27	14	13	
TNM stage				
I and II	41	25	16	0.0125*
III	19	5	14	
Distant metastasis				
No	43	25	18	0.0449*
Yes	17	5	12	
Tumor size (cm)				
≤ 3	25	17	8	0.0184*
> 3	35	13	22	
N stage				
N0	38	22	16	0.108
N1‒3	22	8	14	

### circPOLA2 triggers BC cell malignant phenotype

**Fig. 2:**
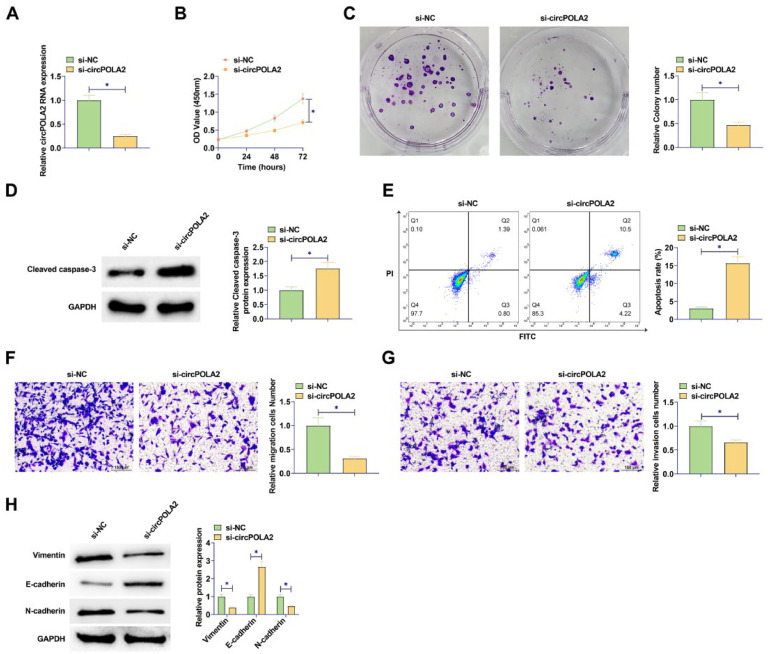
circPOLA2 promotes BC cell malignancy. (A) RT-qPCR measured circPOLA2 after interference; (B) MTT assay detected cell proliferation; (C) Colony formation assay analyzed clonal formation of BC cells; (D) Flow cytometry detected apoptosis rate; (E) Western Blot detected Cleaved caspase-3 protein expression; (F‒G) Transwell detected cell migration and invasion ability. (H) Western blot analyzed Vimentin, E-cadherin, and N-cadherin. Data are expressed as mean ± SD (*n* = 3), * *p* < 0.05.

si-circPOLA2 was designed to interfere with circPOLA2 expression in BC cells. circPOLA2 expression of MCF-7 cells was decreased after si-circPOLA2 transfection, indicating significant transfection efficiency of si-circPOLA2 (Fig. 2A). MTT and colony formation assays indicated that BC cell proliferative capacity was weakened, and the number of cell colonies decreased after circPOLA2 knockdown (Fig. 2B‒C). Flow cytometry results found an apoptosis increase after circPOLA2 knockdown (Fig. 2D). Knockdown of circPOLA2 significantly inhibited the protein expression of Cleaved caspase-3 in MCF-7 cells (Fig. 2E). Transwell assay found that circPOLA2 knockdown inhibited BC cell migratory and invasive capacities (Fig. 2F‒G). Meanwhile, Western blot analysis revealed that knocking down circPOLA2 enhanced E-cadherin and suppressed Vimentin and N-cadherin proteins (Fig. 2H).

### circPOLA2 directly interacts with miR-1224–5p in cells

**Fig. 3:**
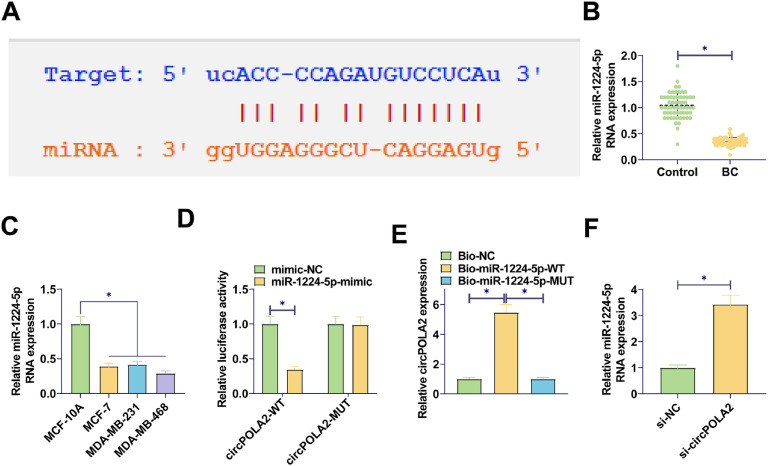
circPOLA2 directly interacts with miR-1224–5p (A) Bioinformatics prediction of target binding sites between circPOLA2 and miR-1224–5p; (B‒C) RT-qPCR measured miR-1224–5p in BC tissues and cell lines; (D) Dual-luciferase reporter gene experiment verified the interaction between miR-1224–5p and circPOLA2; (E) RNA pull-down assay verified the targeting relationship between miR-1224–5p and circPOLA2; (F) RT-qPCR measured miR-1224–5p Data are expressed as mean ± SD (*n* = 3), * *p* < 0.05.

To identify the potential mechanism by which circPOLA2 regulates the phenotype of BC cells, complementary binding sites between miR-1224–5p and circPOLA2 were predicted by Starbase (Fig. 3A). RT-qPCR data demonstrated a decrease in miR-1224–5p expression in BC tissues and cell lines (Fig. 3B‒C). Luciferase activity measurements revealed the inhibition of luciferase activity in WT-circPOLA2 after miR-1224–5-mimic interference, verifying the interaction between circPOLA2 and miR-1224–5p (Fig. 3D). This targeting relationship was additionally confirmed in BC cells by RNA pull-down assay (Fig. 3E). Moreover, si-circPOLA2 forced miR-1224–5p expression in BC cells (Fig. 3F).

### Depleting miR-1224–5p reverses si-circPOLA2-mediated inhibition of malignant behaviors of BC cells

**Fig. 4:**
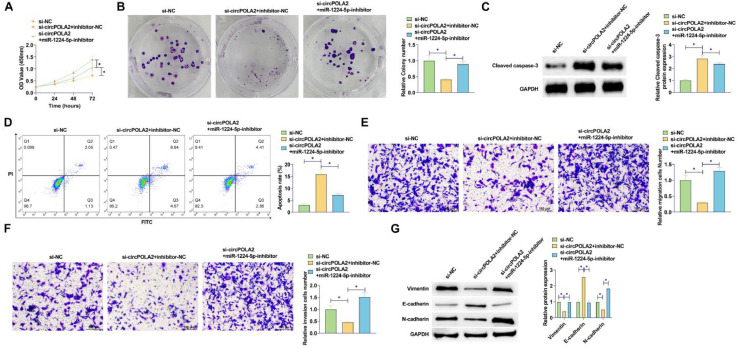
Depleting miR-1224–5p abolishes si-circPOLA2-mediated inhibition of malignant behaviors **of BC cells.** (A) MTT assay detected cell proliferation; (B) Colony formation assay analyzed clonal formation of BC cells; (C) Flow cytometry detected apoptosis rate; (D) Western Blot detected Cleaved caspase-3 protein expression; (E‒F) Transwell detected cell migration and invasion ability. (G) Western blot analyzed Vimentin, E-cadherin, and N-cadherin. Data are expressed as mean ± SD (*n* = 3), * *p* < 0.05.

miR-1224–5p silencing experiments were performed in si-circPOLA2-transfected cells. By reducing miR-1224–5p levels, circPOLA2 knockdown had a weaker repressive effect on cell proliferation (Fig. 4A). The number of colonies of cells co-transfected with miR-1224–5p-inhibitor and si-circPOLA2 increased significantly. It was further confirmed that the suppressive influence of circPOLA2 knockdown on cell proliferation was abolished by miR-1224–5p inhibitor (Fig. 4B). si-circPOLA2 promoted apoptosis of MCF-7 cells, but this apoptosis trend was reduced in cells co-transfected with miR-1224–5p inhibitor (Fig. 4C). Western Blot assay showed that down-regulation of miR-1224–5p significantly inhibited the protein expression of Cleaved caspase-3 (Fig. 4D). miR-1224–5p inhibitor abolished the effects of circPOLA2 knockdown on cell migration and invasion (Fig. 4E‒F). si-circPOLA2 forced E-cadherin and decreased Vimentin and N-cadherin. However, miR-1224–5p inhibitor reduced the effect of circPOLA2 silencing on the above EMT-related factors (Fig. 4G).

### circPOLA2 up-regulates HMGA2 expression by targeting miR-1224–5p

**Fig. 5:**
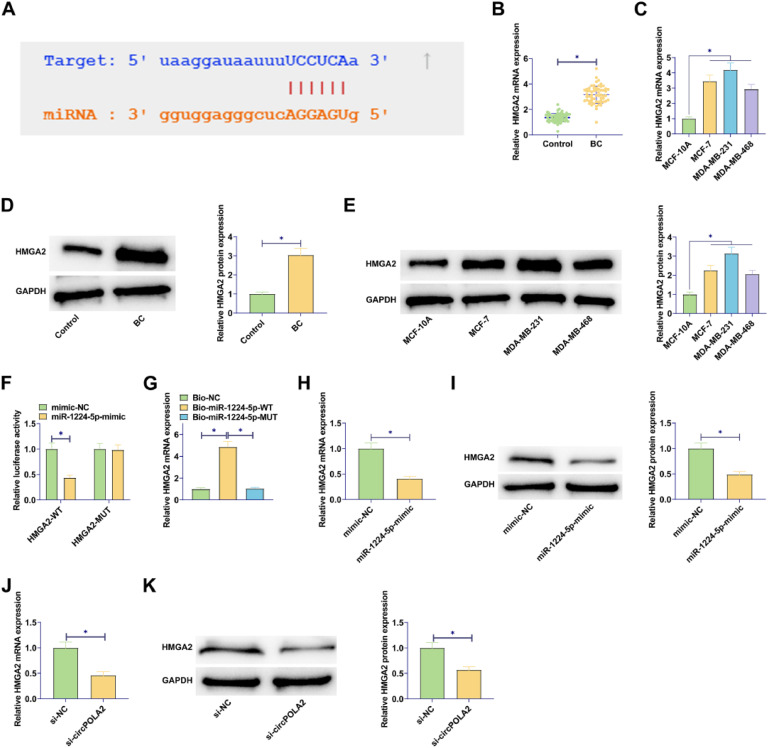
circPOLA2 up-regulates HMGA2 expression by targeting miR-1224–5p (A) Bioinformatics prediction of target binding sites between HMGA2 and miR-1224–5p; (B‒E) RT-qPCR and Western blot measured HMGA2 in BC tissues and cell lines; (F) Dual-luciferase reporter gene experiment verified the interaction between HMGA2 and miR-1224–5p; (G) RNA pull-down assay verified the targeting relationship between HMGA2 and miR-1224–5p; (H‒K) RT-qPCR and Western blot measured HMGA2. Data are expressed as mean ± SD (*n* = 3), * *p* < 0.05.

Complementary binding sites between miR-1224–5p and HMGA2 were predicted by Starbase (Fig. 5A). In addition to this, both miRBase and DIANA-miRPath looked for interactions between miR-1224–5p and HMGA2. miRBase showed that miR-1224–5p targets NM_003483 (HMGA2). miRBase showed that miR-1224–5p interacts with HMGA2 with an Interaction Score of 0.93. RT-qPCR and Western blot results confirmed the elevated expression of HMGA2 in BC tissues and cell lines (Fig. 5B‒E). A dual luciferase reporter gene assay was conducted, presenting that miR-1224–5p-mimic decreased WT-HMGA2 luciferase activity but did not change MUT-HMGA2 luciferase activity (Fig. 5F). Further, RNA pull-down assay confirmed the relationship between HMGA2 and miR-1224–5p (Fig. 5G). miR-1224–5p-mimic transfection in MCF-7 cells inhibited HMGA2 expression (Fig. 5H‒I). At the same time, si-circPOLA2 also down-regulated HMGA2 expression in BC cells (Fig. 5J‒K).

### circPOLA2 mediates BC malignant behavior by HMGA2 regulation

**Fig. 6:**
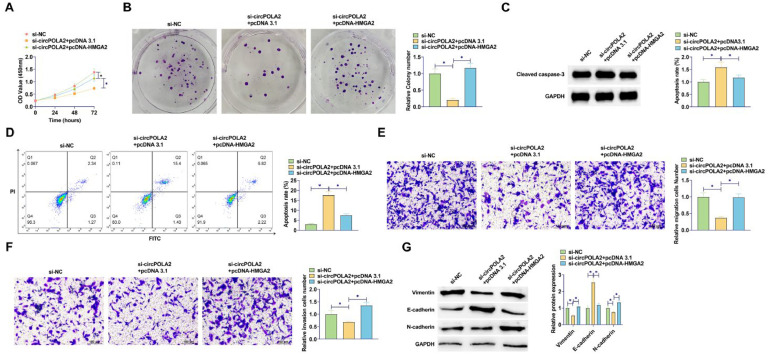
circPOLA2 mediates BC cell malignant behaviors by regulating HMGA2. (A) MTT assay detected cell proliferation; (B) Colony formation assay analyzed clonal formation of BC cells; (C) Flow cytometry detected apoptosis rate; (D) Western Blot detected Cleaved caspase-3 protein expression; (E‒F) Transwell detected cell migration and invasion ability. (G) Western blot analyzed Vimentin, E-cadherin, and N-cadherin. Data are expressed as mean ± SD (*n* = 3), * *p* < 0.05.

To further investigate whether circPOLA2 can regulate HMGA2 through miR-1224–5p, rescue experiments were implemented. HMGA2 overexpression reduced the suppressive influence of circPOLA2 knockdown on cell proliferation (Fig. 6A). The number of cell colonies increased after HMGA2 overexpression, which further confirmed that the suppressive influence of circPOLA2 knockdown on cell proliferation was abolished by HMGA2 overexpression (Fig. 6B). Suppressing circPOLA2 promoted apoptosis of MCF-7 cells, but this apoptosis trend was reduced in cells overexpressing HMGA2 (Fig. 6C). Western Blot assay showed that HMGA2 overexpression significantly inhibited the protein expression of Cleaved caspase-3 (Fig. 6D). HMGA2 overexpression abolished the effects of circPOLA2 knockdown on cell migratory and invasive phenotypes (Fig. 6E‒F). si-circPOLA2 transfection upregulated E-cadherin and lowered Vimentin and N-cadherin proteins, but HMGA2 overexpression impaired the effect of circPOLA2 silencing on these factors (Fig. 6G).

### circPOLA2 enhances the growth of BC cell xenograft tumors in vivo

**Fig. 7:**
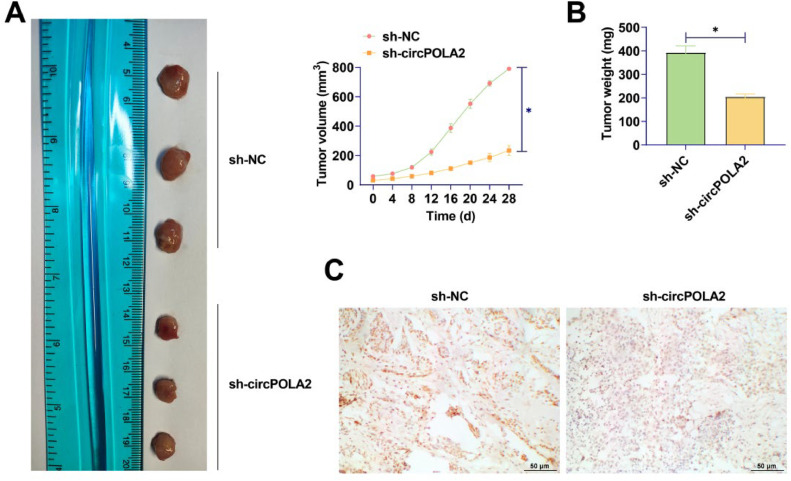
circPOLA2 enhances the growth of BC cell xenograft tumors in vivo. (A) Growth curve of tumor tissue in nude mice; (B) Weight of tumor in nude mice; (C) IHC staining of HMGA2 expression in xenograft tumors. Data are expressed as mean ± SD (*n* = 3), * *p* < 0.05.

MCF-7 cells transfected with si-circPOLA2 or si-NC were injected into nude mice. The mean tumor weight and volume after silencing circPOLA2 were lower (Fig. 7A‒B), and silencing circPOLA2 inhibited BC tumor growth. IHC staining of xenograft tumors showed that HMGA2 expression was decreased after silencing circPOLA2 (Fig. 7C).

## Discussion

BC greatly harms female health worldwide. However, despite improvements in prevention, early diagnosis, and individualized treatment, the prognosis continues to be poor due to recurrence, distant metastasis, and resistance to chemotherapy. There is an urgent need to study the molecular pathogenesis of BC to find its potential targets and provide new strategies for BC. This study found that circPOLA2 was a carcinogenic factor that regulates BC metastasis, and circPOLA2 promoted EMT and metastasis of BC by targeting HMGA2 through sponge miR-1224–5p This suggests that circPOLA2 can be a potential target for BC treatment.

In BC, many circRNAs function as oncogenes in the tumor microenvironment, such as circRNA_DCAF6^29^ and circ_0084927^30^ Similarly, circPOLA2 is an oncogenic factor in lung and cervical cancers as previously described, suggesting that circPOLA2 dysregulation plays an important role in the malignant development of cancer. In the present study, circPOLA2 expression was upregulated in BC. High expression of circPOLA2 was positively correlated with TNM stage, tumor size, and recurrence/distant metastasis in BC. In addition, circPOLA2 expression was similarly upregulated in BC cell lines. To the best of our knowledge, the present study is the first to report that circPOLA2 is upregulated in BC tissues. Thus, the authors explored the mechanism by which circPOLA2 promotes BC tumorigenesis. Silencing circPOLA2 inhibited BC cell growth and metastasis. Western blot showed that silencing circPOLA2 inhibited EMT expression in BC. In addition, silencing circPOLA2 was shown to inhibit tumor growth in vivo experiments. These results all suggest that circPOLA2 is involved in metastasis and malignant progression of BC and may be a biomarker for the diagnosis and prognosis of BC. These results suggest that circPOLA2 has generalized biological significance in cancer. In addition, it has also been shown that circPOLA2 interacts with the protein to affect non-small cell lung cancer^31^ This suggests that circPOLA2 acts through different mechanisms in different cancers, and it is possible that its function is environmentally dependent. circPOLA2′s function needs to be explored in depth. circRNAs can compete with miRNA to regulate gene expression, thereby inhibiting the effect of miRNA on mRNA degradation^32^ This mechanism is involved in regulating the biological behaviors of various tumors^33^, ^34^, ^35^, ^36^ Therefore, circRNAs are promising diagnostic biomarkers and potential therapeutic targets. miR-1224–5p was one of the targets of circPOLA2 through starBase 3.0 prediction. Studies have demonstrated that the upregulation of miR-1224–5p inhibits drug resistance in glioma to suppress cancer development^37^ or inhibits proliferation and invasion of ovarian cancer by targeting SND1^23^ miR-1224–5p is also involved in EMT in tumor cells. miR-1224–5p has been shown to inhibit EMT in laryngeal tumor cells and is closely associated with patient prognosis^38^ These data demonstrate that miR-1224–5p has a broad regulatory role in a wide range of tumors and affects tumor development. The present study further demonstrated that inhibition of miR-1224–5p reversed the inhibitory effect of silencing circPOLA2 on the malignant progression of BC cells. These results suggest that circPOLA2 can target and regulate miR-1224–5p, thereby participating in BC development. This is largely consistent with previous studies of circPOLA2 and cancer progression, further supporting the molecular mechanism of circPOLA2 sponging miRNAs to promote tumor growth and progression by regulating cancer cell proliferation, apoptosis, and migration.

**Fig. 8:**
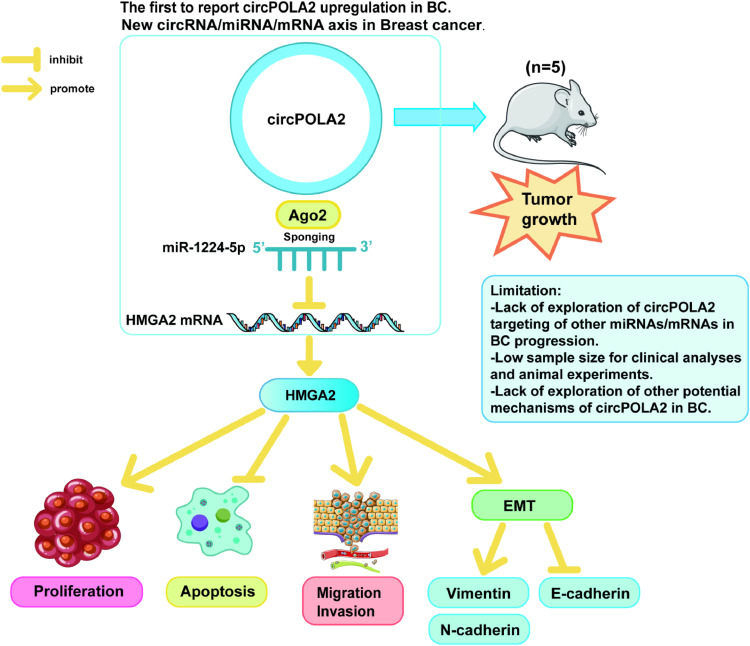
Action of circPOLA2 regulating miR-1224–5p/HMGA2 axis and its effects on BC cell proliferation, apoptosis, migration and invasion, and EMT, illustrating the strengths and limitations of the experiments in this study.

This study confirmed miR-1224–5p's targeting relationship with HMGA2. Previous papers have illustrated that HMGA2 is a tumor promoter in BC^39^^,^^40^ Based on this, this study further discovered that silencing circPOLA2 inhibited the malignant progression of BC cells, but overexpressing HMGA2 impaired the effect of circPOLA2 silencing. Importantly, ectopic expression of HMGA2 in epithelial cells induces EMT, which causes epithelial cells to lose cell polarity and attenuates intercellular adhesion, an important feature of tumor cell metastasis. It has been shown that HMGA2 can promote BC cell proliferation, migration, and invasion by promoting EMT changes^27^ Similar results were obtained in the present study, where HMGA2 overexpression increased E-cadherin and decreased the expression levels of N-cadherin and Vimentin. These results suggest that circPOLA2 competitively binds to miR-1224–5p to regulate HMGA2 expression, promoting BC cell proliferation, migration, invasion, and EMT, and accelerating the development of BC (Fig. 8).

However, there are still some limitations in this study. In this study, the authors did not use bioinformatics methods for circRNA sequencing or microarray screening experiments, and the expression, binding roles, and distribution of circPOLA2 and miR-1224–5p in BC were not further confirmed. Moreover, circPOLA2 acts through a complex network of endogenous RNA competition, whereas the authors only observed the importance of the circPOLA2/miR-1224–5p/HMGA2 axis. Therefore, several potential miRNAs and mRNAs targeted by circPOLA2 during BC progression should be investigated. More importantly, it is difficult to determine the regulation of the circPOLA2/miR-1224–5p/HMGA2 axis on the function of BC cells based on the experiments in this study alone. These deficiencies are the focus of future explorations and will be verified in future research endeavors in a variety of cell lines and animal experiments with a large number of clinical samples and diverse experimental approaches to increase their credibility.

## Conclusion

In summary, silencing circPOLA2 as a molecular sponge of miR-1224–5p down-regulates GIGYF1 expression and inhibits BC cell proliferation, migration and invasion, thereby retarding tumor growth in vivo. Therefore, the circPOLA2/miR-1224–5p/HMGA2 axis was used as a potential target for BC treatment and prognosis, which may become a new target for the treatment and diagnosis of BC, and provide highlights for BC treatment.

## Declaration of competing interest

The authors declare no conflicts of interest.
